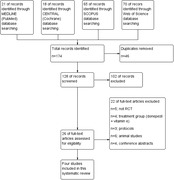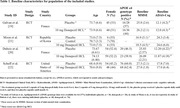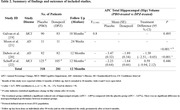# MRI Hippocampus Differential Response to Donepezil vs Placebo in Mild Cognitive Impairment and Alzheimer’s Disease: A Systematic Review of Four Randomized Clinical Trials

**DOI:** 10.1002/alz.087120

**Published:** 2025-01-09

**Authors:** Youssef A. Ismail, Youssef Haitham, Mohammad Walid, Hazim Mohamed, Youssef M. Abd El‐Satar

**Affiliations:** ^1^ Faculty of Medicine Port Said Univeristy, Egypt, Port Said, Port Said Egypt; ^2^ STEM Neurology & Neuropsychological0 Research Group Egypt (SNRGE), Port Said, Port Said Egypt; ^3^ Faculty of Medicine Suez Canal Univeristy, Egypt, Ismailia, Ismailia Egypt; ^4^ Faculty of Medicine, Arish University, Arish, North Sinai Egypt; ^5^ Faculty of Medicine Helwan University, Cairo, Helwan Egypt; ^6^ Faculty of Medicine Cairo Univeristy, Egypt, Cairo, Cairo Egypt

## Abstract

**Background:**

Donepezil, an acetylcholinesterase inhibitor (AChEI), is an FDA‐approved drug to treat these neurodegenerative diseases, e.g., Alzheimer’s Disease (AD) and Mild Cognitive Impairment (MCI). AChEIs are able to stabilize or slow decline in cognition, function, and behavior. Our objective is to investigate whether Donepezil is able to significantly reduce the rate of hippocampal (Hip) atrophy in neurodegenerative diseases.

**Methods:**

We followed the PRISMA statement guidelines during the preparation of this systematic review. We searched in MEDLINE (PubMed), CENTRAL (Cochrane Library, November 2023), SCOPUS, and Web of Science and included randomized clinical trials (RCTs) comparing 10 mg donepezil‐treated with donepezil‐untreated (placebo) and/without control in terms of magnetic resonance imaging (MRI) follow up visits’ results.

**Results:**

A total of four studies out of 174 met our inclusion criteria (599 participants; donepezil = 281, placebo = 318), two of them were ADs and the others were MCIs. 323 participants were female (representing 53.92% of included study population). Follow up between baseline and endpoint results was 12 months. Available outcome data cover reduction of hippocampal atrophy rate in patients with neurodegenerative diseases, but data on several outcome dimensions were either unavailable or not consistently reported across all studies. Results concluded from studies been conducted on MCI patients were statistically insignificant (*P* > 0.05) annual percentage of change (APC) of Hip volume at 12 months compared to placebo, but studies on AD patients indicated statistically significant APC of Hip volume at 24 and 50 weeks (*P* < 0.001), but one of these studies also reported no significant difference in neuropsychological performance between treatment groups.

**Conclusion:**

The findings of this review suggest that donepezil reducing hippocampal atrophy rate was statistically insignificant for MCI and statistically significant for AD, but its clinical significance is questionable until further investigations. It is also important to note that while the data provided insights into the impact of donepezil, there were limitations, such as incomplete reporting of outcome dimensions in some studies.